# Is the Rise in the Prevalence of Renal Replacement Therapy at Older Ages the Price for Living Longer?

**DOI:** 10.3389/fpubh.2018.00138

**Published:** 2018-05-04

**Authors:** Frederik Peters, Christina Westphal, Anneke Kramer, Ronny Westerman

**Affiliations:** ^1^Department of Sociology and Demography, University of Rostock, Rostock, Germany; ^2^Max Planck Institute for Demographic Research, Rostock, Germany; ^3^Project Group Extracorporeal Immunomodulation, Fraunhofer Institute for Cell Therapy and Immunology (IZI), Rostock, Germany; ^4^ERA-EDTA Registry, Department of Medical Informatics, Academic Medical Center, Amsterdam University, Amsterdam, Netherlands; ^5^Competence Center Mortality Follow-Up, Germany National Cohort, Federal Institute for Population Research, Wiesbaden, Germany

**Keywords:** life expectancy, renal replacement therapy, end-stage renal disease, elderly population, Europe

## Abstract

**Background:**

Renal replacement therapy (RRT) is one of the most expensive in renal medicine. Cross-sectional studies suggest that life expectancy increases in the general population are associated with a higher burden of RRT. This study tests this hypothesis in a prospective setting among people aged 75+ living in Western Europe.

**Methods:**

We gathered sex-specific data for 11 Western European countries in 2005–2014. RRT prevalence on country level was extracted from the ERA-EDTA registry, while data on population size and life expectancy for the 75+ age group came from the Eurostat database. GDP per capita was extracted from the OECD database. To measure the association between RRT prevalence and life expectancy, we performed Poisson regression models separately for each country and for all countries combined. To adjust for confounding, GDP per capita as well as time and country-fixed effects were included.

**Results:**

Our analysis revealed that living longer coincides with rising RRT prevalence at ages 75+ in Western Europe between 2005 and 2014. On average, a 1-year increase in life expectancy was associated with a roughly 20% increase in RRT prevalence [(95% CI) 21–23% in men and 19–22% in women]. However, after adjustments for confounding were made, the association became insignificant among women and became weaker among men, falling to a level of 11% [(95% CI) 6–17%].

**Conclusion:**

Living longer was not necessarily associated with a higher burden of RRT in Western European countries.

## Introduction

Life expectancy has been rising rapidly throughout the world (1). But as people are living longer, chronic disease burdens and health-care expenditures have also been increasing (2). To understand this dynamic, it is useful to examine populations with chronic diseases, and especially those with serious conditions such as end-stage renal disease (ESRD). ESRD patients require lifelong renal replacement therapy (RRT). One of the most expensive in renal medicine, RRT already consumes a considerable proportion of national healthcare budgets (3, 4). Globally, around three million patients are currently receiving RRT, and this number is expected to increase to between 5 and 10 million by 2030 (5). The ongoing epidemics of obesity, diabetes, and hypertension are likely to aggravate this problem (6).

Previous studies have suggested that a greater RRT burden might be an inevitable consequence of living longer (7, 8). Increased survival among the general population has been shown to coincide with lower mortality rates among dialysis patients (9). Even at constant incidence rates, the size of the RRT population may be expected to increase simply because dialysis patients are living longer (8, 10). The latest findings indicate that there is a strong direct correlation between life expectancy and RRT prevalence at the country level (5). However, such cross-country correlations are prone to confounding due to unmeasured differences between the objects studied, such as differences in the institutional settings or in patient characteristics. Thus, it is unclear whether living longer really increases the demand for RRT.

Our aim in this study is to determine the relationship between life expectancy gains in the general population and rising RRT prevalence using the latest data from harmonized registries.

We focus on people aged 75+ living in Western Europe for several reasons: first according for redefining the age group for the elderly, the reference of 75 years and older is much more plausible while the phenomenon of “rejuvenation” is more common in younger age groups below 75 years and older. Also there are delayed physical impairments compared with same aged peers 10–20 years ago (11). Second there is an expansion of older dialysis populations across the globe. The increase in the older dialysis population can be mostly attributed to increasing health care access in the population 75 years and older (12). As an example, the Dialysis Outcome and Practice Patterns Study (DOPPS) has shown that nearly half of the dialysis patients in Belgium are older than 75 years (13). Third, it has been demonstrated that older RRT patients are particularly sensitive to changes in general mortality conditions, since they tend to suffer from severe comorbidities in addition to ESRD (14–17).

There is a considerable heterogeneity in remaining life expectancy and RRT procedure preferences among elderly patients (18). This condition impedes the identification of an optimal balance between risks and benefits from best practice intervention on individual level.

We choose to focus on Western European countries, which have highly developed welfare states and aging populations. Moreover, current trends in advanced countries may be indicative of future trends in less developed countries (19). By using a panel regression approach, we are able to analyze the study variables in a prospective country-specific perspective, while adjusting for potentially confounding unobserved country differentials and common time effects.

## Materials and Methods

### Data

In this study, we examined men and women aged 75+ living in 11 European countries (Austria, Belgium, Denmark, Finland, Greece, Iceland, the Netherlands, Norway, Spain, Sweden, and the UK) using register-based data for the years 2005–2014. For our analysis, we used information on RRT, accounted as prevalence per million population (pmp) from the Renal Association-European Dialysis and Transplant Association (ERA-EDTA) registry. The mission of ERA-EDTA Registry was to combine data on RRT prevalence at an annual basis *via* national and regional registries from different European countries which allowed us to compare country-specific effects. Additional information on individual level but not on country level was available for the patient’s date of birth, gender, cause of renal failure, comorbidity status, date of start of first RRT, history of RRT with dates and changes of modality, treatment center, date and cause of death, and information concerning transfer from or to other renal registries. RRT prevalence in this study involved all different types of renal treatment including dialysis (hemodialysis and peritoneal dialysis), hemofiltration, and hemodiafiltration, known as various ways of filtration of blood with or without machine. RRT prevalence also included kidney transplantation, which is the ultimate form of replacement in that the old kidney is replaced by a donor kidney.

On country perspective Belgium, Spain, and the UK had contributed regional-level data, and the other eight countries had contributed national-level data to the ERA-EDTA. Further details on this database were available in the most recent annual report (ERA-EDTA Registry 2016) (20).

The registry provided 100% coverage for almost all of the regions/countries we examined in this study. An exception is Spain, for which we had aggregated information for 10 regions (Andalusia, Aragon, Asturias, Basque Country, Cantabria, Castile/Leon, Castile-La Mancha, Catalonia, Extremadura, and Valencia) covering around 68% of the Spanish population. Data on the population size at each age between 75 and 100, and data on remaining life expectancy at age 75, were extracted from the Eurostat database (21, 22).

Using these data, we calculated the mid-year population size for each region. Information on GDP per capita in purchasing power standard US$ at constant prices (2010) was extracted from the OECD database [OECD (23)].

### Statistical Analysis

Our dependent variable was RRT prevalence in pmp for each year in period 2005–2014, including patients on dialysis (hemodialysis and/or peritoneal dialysis), hemofiltration, and hemodiafiltration and with kidney transplantation. Remaining life expectancy at age 75 was served as the independent variable. Using R version 3.1.1., we performed Poisson regression analyses to assess the within-country correlation between RRT prevalence and life expectancy at age 75 over time. First, we computed bivariate associations between the two variables separately for each country with no adjustments (Model 1). Second, to estimate the average effect with country-fixed effects, we computed the bivariate associations between the two variables for all countries combined (Model 2). Third, we computed multivariate models to assess for the association between the two variables and we have adjusted for country, year and log GDP (per capita) (Model 3).

Generally, these models (Models 1–3) related the log RRT prevalence with the log mid-year population as offset to a linear combination of parameters. The exponent of the parameter coefficients provided the relative risk; i.e., the proportional change in RRT prevalence given a 1-year change in life expectancy. All models were run using a performing Poisson regression. We captured for confounding in the models while country-specific variation in treatment procedure, health-care expensive and on country level could influence the statistical association between life expectancy and RRT prevalence.

## Results

From 2005 to 2014, RRT prevalence and life expectancy in the general population increased in all of the countries studied. The RRT prevalence rose 32.8% (Denmark) to 120% (Iceland) among men and 25.9% (Finland) to 99.8% (Netherlands) among women (Table 1), while life expectancy in the general population increased 7.3% (Iceland) to 18.4% (Spain) among men and 4.7% (Sweden) to 13.0% (Belgium) among women. There was a general upward trend in GDP per capita over the period in most of the considered countries. However, while GDP per capita increased by around 7% in Austria and in Sweden, it declined 5% in Spain and 19% in Greece.

**Table 1:** Descriptive statistics of the study variables in 2005 and 2014 by sex and country.

Country	Renal replacement therapy prevalence, per million population (age 75+)			Life expectancy (age 75+)			Per capita GDP in US$		
			
	2005	2014	Change (%)	2005	2014	Change (%)	2005	2014	Change (%)
**Males**									
Austria	566	966	70.7	10.2	11.7	14.7	39,945	42,765	7.1
Belgium	1,349	2,536	88.0	9.8	11.5	17.3	38,114	39,596	3.9
Denmark	372	494	32.8	9.6	11.1	15.6	42,736	41,994	−1.7
Finland	262	442	68.7	10.2	11.3	10.8	37,619	37,490	−0.3
Greece	1,314	2,275	73.1	10.2	11.9	16.7	29,822	24,286	−18.6
Iceland	10	22	120.0	11	11.8	7.3	39,012	40,954	5.0
Netherlands	880	1,765	100.6	9.7	11.3	16.5	42,797	44,603	4.2
Norway	357	516	44.5	10.2	11.4	11.8	59,402	59,951	0.9
Spain	2,789	4,918	76.3	10.3	12.2	18.4	32,760	31,181	−4.8
Sweden	731	1,028	40.6	10.4	11.5	10.6	40,088	42,806	6.8
UK	3,356	5,995	78.6	10.3	11.6	12.6	36,555	37,795	3.4

**Females**									
Austria	503	660	31.2	12.3	13.7	11.4	39,945	42,765	7.1
Belgium	1,139	1,722	51.2	12.3	13.9	13.0	38,114	39,596	3.9
Denmark	204	288	41.2	11.9	13	9.2	42,736	41,994	−1.7
Finland	185	233	25.9	12.9	13.6	5.4	37,619	37,490	−0.3
Greece	962	1,443	50.0	11.8	13.3	12.7	29,822	24,286	−18.6
Iceland	6	11	83.3	13	14.1	8.5	39,012	40,954	5.0
Netherlands	613	1,225	99.8	12.3	13.5	9.8	42,797	44,603	4.2
Norway	157	264	68.2	13	13.5	3.8	59,402	59,951	0.9
Spain	2,230	3,417	53.2	12.9	15	16.3	32,760	31,181	−4.8
Sweden	398	510	28.1	12.9	13.5	4.7	40,088	42,806	6.8
UK	1,940	3,434	77.0	12.1	13.4	10.7	36,555	37,795	3.4

A statistically significant positive within-country association between RRT prevalence and life expectancy was detected among both men and women in all of the countries studied, except in Iceland and among women in Finland (Table 2).

**Table 2:** Relative effect of a 1-year change in life expectancy at age 75 on the renal replacement therapy prevalence at ages 75+ (*N* = 110).^c^

Model	Country	Males RR (95% CI)	Females RR (95% CI)
Model 1 (unadjusted)	Austria	**1.23** (1.17–1.30)	**1.18** (1.11–1.26)
	Belgium	**1.29** (1.25–1.33)	**1.21** (1.17–1.26)
	Denmark	**1.07** (1.00–1.14)	**1.25** (1.13–1.39)
	Finland	**1.20** (1.09–1.32)	1.15 (0.97–1.37)
	Greece	**1.16** (1.13–1.19)	**1.11** (1.07–1.15)
	Iceland	1.19 (0.84–1.69)	1.48 (0.82–2.58)
	Netherlands	**1.30** (1.25–1.34)	**1.63** (1.54–1.73)
	Norway	**1.35** (1.24–1.46)	**1.87** (1.51–2.32)
	Spain	**1.20** (1.18–1.22)	**1.13** (1.11–1.15)
	Sweden	**1.24** (1.16–1.32)	**1.35** (1.17–1.57)
	UK	**1.24** (1.21–1.26)	**1.35** (1.31–1.39)

Model 2^a^ (unadjusted)	all countries	**1.22** (1.21–1.23)	**1.20** (1.19–1.22)

Model 3^b^ (adjusted)	all countries	**1.11** (1.06–1.17)	0.98 (0.94–1.02)

*Values that are significant at a level of at least 5% are in bold*.

*^a^Including country-fixed effects*.

*^b^Including country-fixed and time-fixed effects and GDP per capita*.

*^c^Multiplicity was not accounted for in the analysis*.

According to these estimates, a 1-year increase in life expectancy was associated with an increase in RRT prevalence of 7% (Denmark) to 35% (Norway) among men and of 11% (Greece) to 87% (Norway) among women.

In the model with all countries (Model 2), a 1-year increase in life expectancy was associated with an increase in RRT prevalence of 22% among men and of 20% among women. In the model that adjusted for confounding (Model 3), this effect has declined to 11% among men, and is no longer statistically significant among women.

## Discussion

### Major Findings

Our analysis revealed that between 2005 and 2014, rising longevity coincided with an increase in RRT prevalence among people aged 75+ living in Western Europe. On average, a 1-year increase in life expectancy at age 75 was associated with an increase in RRT prevalence of around 20%. However, after adjusting for confounding, we found that this association is much weaker among men and has disappeared among women. Thus, the independent relationship between rising life expectancy in the general population and increasing RRT prevalence was much smaller than it was previously assumed. These results suggested that the ongoing process of extending longevity will not necessarily lead to an increase in the prevalence of severe chronic conditions, such as ESRD.

### Relation to Previous Work

There is growing evidence that improvements in general mortality conditions, and especially the reduction in deaths from cardiovascular diseases, are associated with higher survival rates among RRT patients (9, 24, 25). In response to recent findings for several countries that RRT incidence rates have been stable (17, 26), scholars have argued the improvements in survival could lead to higher RRT prevalence (27). Our main findings provide only limited support for that hypothesis, as we have found a weak independent correlation between life expectancy and RRT prevalence among men, and no correlation among women. These observations seem to challenge arguments made by Liyanage et al. (5) and others that life expectancy in the general population should be added to the list of important predictors of RRT prevalence. Instead, the gaps between the estimates generated by our unadjusted and adjusted models suggest that life expectancy might be viewed as a proxy for other factors that drive RRT prevalence. Therefore, life expectancy might indeed be useful for predicting RRT prevalence in countries where more detailed information is not available. However, researchers should be careful when making causal inferences based on this relationship.

In addition to addressing the question of future demand for RRT, our results contribute to the literature on the relationship between chronic diseases and life expectancy. According to the “failure of success hypothesis” put forward by Gruenberg (28), improving survival conditions may be expected to result in higher numbers of older people with severe chronic conditions. This hypothesis is contradicted by our finding that among women living in Western Europe, rising longevity was not necessarily associated with an increase in RRT prevalence.

### Strengths and Limitations

A major strength of our prospective analysis is our approach to confounding in the relationship between life expectancy and RRT prevalence. Unlike previous cross-sectional studies that compared several countries at a single point in time (5, 29), we tracked changes in life expectancy and RRT prevalence within each country over a decade. This approach enabled us to adjust for confounding due to unobserved country differentials, which is impossible in a cross-country comparison (30). Moreover, analyzing time series of multiple countries allowed us to adjust for unobserved period effects that may have affected the relationship between life expectancy and RRT prevalence. Finally, we minimized the heterogeneity of our sample by focusing on elderly people living in Western Europe, and by analyzing women and men separately. Our results are thus less affected by sample composition changes than the findings of studies that do not take into account the age or sex of RRT patients explicitly.

Our use of high-quality data on mortality and RRT prevalence from harmonized registries is another advantage of our study. Unlike data on the prevalence/incidence of chronic conditions, such as diabetes, hypertension, or chronic kidney disease; our study variables of death and ESRD treatment represent clearly defined endpoints. Half of our sample data come from Nordic countries, which have a long tradition of collecting reliable data in administrative registries. This is, however, also a weakness of our study, as several important Western European countries (e.g., France, Germany, Italy, and Portugal) are not included in our analysis. Thus, our finding that there is only a mild association between life expectancy and RRT prevalence may be related to the dominance in our study of Nordic countries with universal and generous welfare systems that tend to be associated with superior health outcomes (31). However, our sensitivity analysis demonstrated that the central results were robust to the exclusion of the Nordic countries, except for a widening of the confidence intervals, which may have been due to the smaller sample size (Table 3). The same was true after excluding Belgium and Greece, previously described to perform worst in terms of ESRD (32). While this sensitivity analysis confirms the robustness of our central findings, it does not indicate whether the results would change if countries that were excluded due to unavailable data were included.

**Table 3:** Sensitivity analysis of the association between life expectancy and renal replacement therapy prevalence depending on the selection of countries.^b^

Model	Countries	Exclusion	Males RR (95% CI)	Females RR (95% CI)
Model 3^a^ (adjusted)	6	Nordic countries	**1.09** (1.03–1.16)	0.95 (0.90–1.00)
	9	Belgium and Greece	**1.14** (1.07–1.22)	**0.94** (0.89–0.99)

*Values that are significant at a level of at least 5% are in bold*.

*^a^Including country and time-fixed effects and GDP per capita*.

*^b^Multiplicity was not accounted for in the analysis*.

Another possible limitation of our analysis is our use of the onset of RRT as a proxy for ESRD. Guidelines for initiating RRT, which are generally based on glomerular filtration rates and the presence of comorbidities like obesity, diabetes, and hypertensions and also the treatments of these conditions differ across countries, and even have changed over time.

Otherwise theses cross-country variations regarding the interference of comorbidities potentially harm the statistical association between RRT prevalence and life expectancy and even bedevil plausible interpretation.

However, these issues mainly affect the incidence of RRT and are less relevant for the RRT prevalence, the variable we used in our study. Our methodological approach of focusing on within-country variation adjusted for time-fixed effects also mitigates this problem. Ultimately, we believe it is more appropriate to focus on the treatment of ESRD than on its presence. Both health-care costs and patient quality of life are more affected by RRT than by ESRD itself.

Finally, all estimates are performed with a Poisson regression. The results for the negative binomial regression are not shown. Some caution is need while possible overdispersion of the RRT prevalence in the Poisson regression models can result in some biased estimates follow with some alternative interpretation. In that case the negative binomial distribution alternatively should provide a better statistical performance. Reasonably there is no evidence for overdispersion and even Poisson regression models and the negative binomial distribution models show very similar estimates with the same test results.

### Explanation of Results

Our results indicate that among older people in Europe, changes in RRT prevalence and life expectancy, and the association between these two variables, are surprisingly similar. This finding suggests that these trends are driven to a large extent by factors beyond the control of country-specific policies or other characteristics. Yet, recent evidence suggests that health-care funding might be a crucial factor in the increase in both the use of expensive treatments and life expectancy (33, 34). Dialysis is a prime example of a costly medical treatment that has been rationed since it was first introduced, especially among older people (35). Although the gap between the demand for and the supply of RRT is comparatively small in Europe, the time frame analyzed in our study marked a period in which older people were increasingly being included in RRT programs (17, 36). The role of such economic factors in the expansion of RRT at older ages is underscored by our finding that only a weak association between life expectancy and RRT remained after adjusting for GDP and other confounders (37).

A second factor that influences RRT trends independent of changes in life expectancy in the general population is the increasing tendency of physicians to recommend alternatives to RRT, such as palliative care, for older ESRD patients (38). As such decisions are mainly based on the expected survival benefits to the patient, this trend may indicate that the mortality rates of ESRD patients are not improving at the same pace as those of the general population (39). The progress made in the detection and treatment of cardiovascular conditions, which has driven a large share of the rise in life expectancy at older ages, may have helped patients with chronic kidney disease avoid or delay the onset of ESRD (17). Hence, the patients who develop ESRD are increasingly a selected unhealthy subgroup of all patients with chronic kidney disease.

Our findings highlight the importance of taking gender into account when monitoring RRT trends, as changes in life expectancy related to RRT prevalence could be found for men, but not for women. Such sex-specific differences in renal outcomes have been reported before. Compared with their female counterparts, men with chronic kidney disease tend to progress to ESRD more rapidly, and to have a higher mortality rate after the onset of ESRD (40, 41). This may explain why RRT prevalence is particularly sensitive to improvements in mortality conditions among male patients.

### Relevance for the Research Field and Policy Makers

This is the first study that had related changes in life expectancy in the general population to changes in RRT within a prospective setting. Our findings provided new insights for tackling the question of whether increased life expectancy is indeed jeopardizing the financial stability of health-care systems (42). When we look at women, who tended to experience mortality reductions before men, we saw that living longer was not associated with increased RRT prevalence. Life expectancy at age 75 in European countries was projected to increase by around 1 year by 2030 (43). Our estimates indicated that over this period RRT prevalence may rose up to 17% among men and 6% among women (based on the upper level of the CIs of our central estimates in Model 3, Table 2). The results of our study, which had examined a region with high levels of population aging and economic prosperity, maybe provided a benchmark for developing countries where the demand for RRT and life expectancy in the general population were currently increasing rapidly (5). As physicians were increasingly able to prevent the progression to ESRD and were prescribing more conservative treatments for ESRD, the burden of RRT might decline even further in both developed and developing countries. The partial decline in incidence rates and the stabilization of RRT prevalence rates among older people in Western Europe might be early indications that the prospects for renal patients are improving (35).

## Conclusion

An increase in the RRT burden was not necessarily linked to longer lifespans at older ages. Thus, concerns that RRT prevalence will rise sharply in the future might turn out to be too pessimistic.

## Author Contributions

FP and RW are responsible for the conception or design, or analysis and interpretation of data; FP, RW, and CW are responsible for drafting the article or revising it. AK and CW have provided intellectual content of critical importance to the work described. All four authors are responsible for the final approval of the version to be published.

## Conflict of Interest Statement

The results presented in this study have not been published previously in whole or part, except in abstract form. We have no conflicts of interest to disclose.
